# Nutritional interventions to augment resistance training-induced skeletal muscle hypertrophy

**DOI:** 10.3389/fphys.2015.00245

**Published:** 2015-09-03

**Authors:** Robert W. Morton, Chris McGlory, Stuart M. Phillips

**Affiliations:** Exercise Metabolism Research Group, Department of Kinesiology, McMaster UniversityHamilton, ON, Canada

**Keywords:** muscle protein synthesis, strength, protein balance, leucine, whey, anabolism

## Abstract

Skeletal muscle mass is regulated by a balance between muscle protein synthesis (MPS) and muscle protein breakdown (MPB). In healthy humans, MPS is more sensitive (varying 4–5 times more than MPB) to changes in protein feeding and loading rendering it the primary locus determining gains in muscle mass. Performing resistance exercise (RE) followed by the consumption of protein results in an augmentation of MPS and, over time, can lead to muscle hypertrophy. The magnitude of the RE-induced increase in MPS is dictated by a variety of factors including: the dose of protein, source of protein, and possibly the distribution and timing of post-exercise protein ingestion. In addition, RE variables such as frequency of sessions, time under tension, volume, and training status play roles in regulating MPS. This review provides a brief overview of our current understanding of how RE and protein ingestion can influence gains in skeletal muscle mass in young, healthy individuals. It is the goal of this review to provide nutritional recommendations for optimal skeletal muscle adaptation. Specifically, we will focus on how the manipulation of protein intake during the recovery period following RE augments the adaptive response.

## Introduction

Beyond its role in locomotion, skeletal muscle is the largest site of postprandial glucose disposal, a large site of lipid oxidation, and a substantial contributor to resting metabolic rate (for review see Wolfe, 2006). As a result, considerable research using stable isotopic tracers has been conducted that has aimed to understand the biology of muscle protein turnover in response to various stimuli. What this work has shown us is that the size of human muscle mass is dictated by diurnal changes in rates of muscle protein synthesis (MPS) and muscle protein breakdown (MPB) (Phillips, 2004). In the rested, fasted state, rates of MPB exceed those of MPS and thus skeletal muscle is in a state of negative net protein balance (Biolo et al., 1995b). However, in response to amino acid (AA) or protein feeding, there is a significant but transient increase in rates of MPS and no significant change in MPB rendering skeletal muscle in a state of positive net protein balance (Biolo et al., 1997; Phillips, 2004). It is the relative contribution of these fed and fasted periods to overall net protein balance that dictates skeletal muscle mass homeostasis over time (Phillips, 2004).

In addition to the protein feeding-induced increases in MPS, resistance exercise (RE) also imparts a positive impact on skeletal muscle size (Chesley et al., 1992; Yarasheski et al., 1993; Cermak et al., 2012). Indeed, a single bout of RE in the fasted state significantly increases rates of MPS, however, this rise in MPS is not enough to promote a positive net protein balance (Biolo et al., 1995b). Instead, RE serves to potentiate MPS in response to AA feeding (Biolo et al., 1997), an effect that may persist for up to 24 h (Burd et al., 2011). Therefore, repeated bouts of RE and protein feeding result in skeletal muscle hypertrophy (Cermak et al., 2012). What remains largely unknown is what the most anabolic or sensitizing RE protocol is. Moreover, data pertaining to the optimal dose, timing and quality of protein intake to optimize post-RE muscle anabolism have only recently enabled appropriate recommendations to be made. The aim of this review is to concisely summarize these data as well as discuss new evidence with regards to RE prescription for muscle hypertrophy. We do not provide a comprehensive overview of the cellular and molecular mechanisms regulating cell size but refer the interested reader to other reviews on this topic (Adams and Bamman, 2010; Egan and Zierath, 2013; Blaauw and Reggiani, 2014).

## Protein dose

The first study to examine a protein dose-response relationship with MPS following RE was conducted by Moore et al. (2009). Moore et al. (2009) fed whole-egg proteins after a bout of RE to healthy young men with a wide range of resistance-training experience (4 months to 8 years). The authors found that after a bout of unilateral lower-body RE the MPS response plateaued with ingestion of 20 g of protein such that there was no statistically significant benefit toward MPS with the ingestion of 40 g (Moore et al., 2009). Alternatively phrased, ingestion of 20 g of protein resulted in 89% of the response conferred by ingestion of 40 g. In young, resistance-trained (≥6 months previous weight-lifting experience) men 20 g of whey protein following unilateral RE was also shown to sufficiently stimulate post-absorptive MPS with no further increase ingesting 40 g (Witard et al., 2014a). It appears that 20 g of whey protein (or ~0.25 g protein/kg) is an ample amount of protein to ingest for healthy young men both at rest (Cuthbertson et al., 2005) and after exercise (Moore et al., 2009) regardless of training status (Witard et al., 2014a). Similar results have also been found at rest using whole food (90% lean ground beef) in young men and women where a moderate (~30 g protein) amount was just as effective as a high (~90 g protein) amount at stimulating MPS (Symons et al., 2009). Altogether, these results suggest that 20 g is the maximally effective protein dose per meal in healthy, young individuals. Protein consumed beyond this level is oxidized at a higher rate (Moore et al., 2009; Witard et al., 2014a) and results in urea production (Witard et al., 2014a) indicating there is a limit of AAs that can be used for MPS. The theory behind why, with increasing protein doses, there is a ceiling on MPS has been termed the “muscle full effect” (Atherton et al., 2010). It is important to acknowledge that these dose-response studies have been limited to lower limb RE and thus it remains unknown as to whether the absolute dose of protein required to maximally stimulate rates of MPS following whole-body RE is >20 g.

In this respect, we have refined the estimates for protein to a dose that is expressed per kilogram of body mass or even lean body mass (Moore et al., 2015). Using a two-phase linear regression model we reported that the dose of protein beyond which there was no further increase in MPS in young men was 0.25 g/kg/meal (90% confidence interval 0.18–0.3 g/kg/meal). To account for inter-individual variability we propose the addition of two standard deviations to our estimate, yielding a dose of protein that would optimally stimulate MPS at intake of 0.4 g/kg/meal. In our view, ingestion of protein beyond this dose would result in no further stimulation of MPS. The effects of AA ingestion beyond that needed to maximally stimulate MPS may include metabolic feedback regulation (Layman et al., 2015), satiety (Leidy et al., 2015), and thermogenesis (Acheson et al., 2011). Nonetheless, it needs to be appreciated that AA availability at levels beyond the rate at which they can be used for protein synthesis or other AA-requiring processes means that the amino nitrogen will be used for ureagenesis (Price et al., 1994; Witard et al., 2014a).

Changes in MPS are much greater (4–5 times) in response to stimuli such as contraction and feeding than MPB in healthy humans (Phillips et al., 1997, 2009; Rennie et al., 2004). It has been theorized that defining the protein dose that optimally stimulates MPS is insufficient to accurately characterize the true “anabolic potential” of protein-containing meals (Deutz and Wolfe, 2013). Citing data from whole-body protein turnover Deutz and Wolfe made the case that larger doses of protein can still be more anabolic than smaller doses due to a marked suppression of protein breakdown (Deutz and Wolfe, 2013). The problem in translating these findings to skeletal muscle is that non-muscle tissues dominate whole-body measures of protein turnover, with muscle accounting for only 25–30% of whole body protein turnover (Nair et al., 1988). Thus, even if there is increasingly positive whole-body protein balance with protein doses higher than what we are recommending here we propose that those would be predominantly due to suppression of proteolysis in non-muscle tissues. Even if 25–30% of the suppression of whole-body proteolysis with larger protein doses (Deutz and Wolfe, 2013) were from skeletal MPB such potential gains would be, in our estimation, unlikely to impart a marked benefit in terms of stimulating muscular hypertrophy. While such a conclusion awaits experimental confirmation we propose that marked suppression of proteolysis may not be an optimal strategy to pursue for those engaging in RE. In our opinion, given the multiple mechanisms damaging muscle during exercise, a higher rate of protein turnover (and not persistently suppressing proteolysis) would provide a more efficient mechanism for the removal of damaged proteins.

## Timing of protein ingestion

We have known for some time that RE alone results in a long-lasting elevation in MPS for at least 48 h and MPB for 24 h (Phillips et al., 1997); thus, even in the basal fasted state there is a subsequent increase in the turnover of muscle proteins. RE alone elevating basal MPS will “prime” the muscle to be responsive, in terms of an increased sensitivity of MPS, to aminoacidemia. The duration of this sensitivity is at least 24 h (Burd et al., 2011) and, based on the similar protein dose thresholds (Moore et al., 2009; Witard et al., 2014a), we predict no difference in sensitivity between untrained and trained individuals. Given the sensitizing effect of RE, we conclude it is most advantageous to ingest protein and generate hyeraminoacidemia in the post-RE period.

Some have postulated that pre-exercise protein ingestion may also “prime” the system and offer some advantage over a post-exercise supplementation strategy. However, ingesting 20 g of whey protein either before or 1 h after 10 sets of leg extension resulted in similar rates of AA uptake (Tipton et al., 2007). In other studies there was no benefit shown with pre-exercise AA feeding (Fujita et al., 2009; Burke et al., 2012a). Considering the synergistic response of aminoacidemia following RE (Biolo et al., 1997; Burd et al., 2011), we see it as being optimal to ingest protein immediately following RE. Moreover, we speculate pre-exercise aminoacidemia may blunt the subsequent post-RE MPS response to AAs due to an overlap in the aminoacidemic responses and a muscle full effect (Atherton et al., 2010).

There is only one study to date that has supplemented with protein during exercise and examined the MPS response (Beelen et al., 2008). Beelen and colleagues supplemented young men during an extended RE workout. The supplements were taken before and every 15 min during exercise providing 0.15 g/kg/h carbohydrate with or without 0.15 g/kg/h casein hydrolysate. There was a greater MPS response with carbohydrate plus protein ingestion, which was most likely due to the protein; however, the extra total energy cannot be discounted as a factor (Beelen et al., 2008). Evidence suggests that during-exercise consumption of protein may be beneficial though once again we counsel caution on this practice as the additional post-exercise hyperaminoacidemia may be less effective due to the muscle full effect.

A recent meta-analysis examining protein timing and hypertrophy concluded that the ingestion of a post-exercise supplement in closer temporal proximity to RE positively influenced hypertrophy (Schoenfeld et al., 2013); however, after adjustment for all covariates, the authors concluded that total protein intake was the strongest predictor of muscular hypertrophy and that protein timing did not influence hypertrophy. Nonetheless, practical advice would dictate that the post-exercise period is a time when rehydration, refueling (carbohydrate), and repair (3R) of damaged tissues should occur. We propose that it is still a pragmatic message to tell athletes to ingest fluid, carbohydrates, and protein to accomplish the goals defined by the 3R.

How protein should be consumed throughout the day is matter of debate. In an acute study, an “intermediate” pattern of whey protein ingestion (4 × 20 g every 3 h) throughout a 12 h recovery period post-RE was found to be more effective than ingestion of large boluses (2 × 40 g every 6 h) or a pulse (8 × 10 g every 1.5 h) protocol at stimulating MPS (Areta et al., 2013). These results are in agreement with the muscle full effect where, when AA delivery is sufficient (~20 g), AAs are no longer used for MPS and are targeted for oxidation (Moore et al., 2009; Atherton et al., 2010; Witard et al., 2014a). However, many studies examining the impact of protein feeding on MPS either infuse AAs or provide protein in a bolus form. Though these are an efficient and direct way to provide protein in a laboratory setting, it is not how protein is consumed in the applied setting (i.e., a mixed macronutrient meal). The macronutrient composition and form of meal intake may influence both the meal-induced rise in hyperaminoacidemia and protein synthesis (Burke et al., 2012b). It is also important to, when considering the distribution of protein throughout the day, acknowledge that the recommended dietary allowance for the United States and Canada is 0.8 g/kg/day, which, for an 80 kg individual, would equate to only 64 g of protein per day. Future studies should focus on mixed macronutrients meals and rates of muscle protein turnover over a longer period of time.

Pre-sleep feeding is a time when protein provision may provide a marked benefit to remodel muscle proteins. Ingestion of 40 g of casein protein before bed stimulates MPS and improves net protein balance overnight in healthy young men (Res et al., 2012). Recently, a 12 week progressive RE training study showed that a pre-sleep casein beverage (27.5 g protein, 15 g carbohydrate, 0.1 g fat) in comparison with a placebo beverage augmented muscle mass, muscle fiber area, and strength gains (Snijders et al., 2015). However, the control group in this study did not receive a protein supplement resulting in a 0.6 g/kg difference in total protein intakes (1.3 vs. 1.9 g/kg/d), which some would argue would confer an advantage to the supplemented group regardless of when the protein was consumed. This may be the case and we acknowledge that 1.3 g/kg/d does not fall within even our recommendations for a protein intake that appears to be optimal for hypertrophy (Phillips, 2014a). Nonetheless, it is interesting to note that in a meta-analysis done by Cermak et al. (2012) only 3 of the 16 studies she analyzed showed statistically significant gains in lean mass with protein supplementation in young persons. While there were a further 4–5 studies that approached statistical significance, the fact that only 3 (19%) of the studies [one of which was in women in a hypoenergetic state (Josse et al., 2011)] independently reported augmented hypertrophy with protein supplementation shows that protein's effect on hypertrophy is small compared to the stimulus of the exercise itself. The point we make here is that the magnitude of the effects seen by Snijders et al. (2015) are impressive even considering the extra protein ingested and so we propose that the pre-sleep timing of the protein supplement was as, if not more, important as the higher protein intake of the supplemented group.

Altogether, we propose that the timing of protein intake is an important variable to consider in optimizing skeletal muscle recovery and hypertrophy. It appears optimal to ingest protein in the post-exercise period though the purported “anabolic window” for protein ingestion lasts at least 24 h (Burd et al., 2011) and does not have as drastic an effect on outcomes as has been believed (Schoenfeld et al., 2013). It is also important to ingest protein in sufficient doses (~0.4 g/kg/meal) distributed throughout the day (Areta et al., 2013). Lastly, ingesting AAs in larger doses of protein (40 g casein or up to 0.6 g/kg/meal) pre-sleep appears to augment both acute overnight MPS (Res et al., 2012) and chronic skeletal muscle adaptations (Snijders et al., 2015). We wish to emphasize, however, that the magnitude of gains that are attributable to protein supplementation compared to the overall gains made as a result of the RE training program itself appear to be relatively small.

## Protein quality

There are inherent differences in quality between the three most commonly consumed isolated protein sources: soy, casein, and whey. Proteins such as whey and soy are digested relatively rapidly, resulting in rapid aminoacidemia, and induce a larger but more transient rise in MPS than casein (Tang et al., 2009; Reitelseder et al., 2011). Whole-body protein synthesis is stimulated more with whey protein whereas whole-body protein breakdown is suppressed with ingestion of casein (Boirie et al., 1997). After ingestion of isolated casein, soy and whey protein (all providing 10 g EAA) the acute (3 h) rise in MPS was found to be greatest with whey protein both at rest and following exercise (Tang et al., 2009). Interestingly, soy protein had higher MPS than casein at both rest and after exercise as well (Tang et al., 2009). It appears that at least up to 3 h post-RE the most effective protein source is whey (Tang et al., 2009). Even for those considering weight loss, after 2 week of being hypocaloric, habitual daily consumption of whey (54 g) is more effective than soy at offsetting the decline in the postprandial MPS response (Hector et al., 2015).

In an effort to elucidate the attenuated anabolic response with casein supplementation, we evaluated the rates of MPS after a bout of RE with either a single bolus (25 g) or small pulses every 20 min (2.5 g) of whey protein (West et al., 2011). The 25 g bolus of whey protein lead to higher MPS between both 1–3 and 3–5 h post-exercise (West et al., 2011). The rapid and immediate bolus may be increasing EAA delivery to the muscle, specifically leucine, to a certain threshold that is triggering a MPS and the associated anabolic pathways. Indeed, blends of protein (1:2:1, whey:casein:soy) were later shown, when leucine content was matched, to be as effective as whey in stimulating MPS (Reidy et al., 2013). Furthermore, participants given 25 g of whey protein or 6.25 g whey with 5 g leucine added showed an increased MPS at rest and after RE to a similar extent despite a four-fold lower protein dose (Churchward-Venne et al., 2014). It appears that the leucinemia (and quite possibly the ensuing intramuscular leucine concentration) is the driver of the MPS response and thus the recovery process. The addition of isoleucine and valine (the other branched-chain AAs) does not improve MPS (Churchward-Venne et al., 2014). This response is an underappreciated result considering many supplements contain combinations of the branched-chain AAs, which, based on our data, would not be advantageous to consume co-temporally because they share the same transporter (Hyde et al., 2003). Thus, as we speculated (Churchward-Venne et al., 2014), consumption of crystalline BCAA resulted in competitive antagonism for uptake from the gut and into the muscle and was actually not as effective as leucine alone in stimulating MPS. Despite the popularity of BCAA supplements we find shockingly little evidence for their efficacy in promoting MPS or lean mass gains and would advise the use of intact proteins as opposed to a purified combination of BCAA that appear to antagonize each other in terms of transport both into circulation and likely in to the muscle (Churchward-Venne et al., 2014).

It appears that post-exercise MPS, measured within 3 h, is optimized by protein ingestion that contains a high leucine content where proteins are rapidly digested (i.e., whey) (Tang et al., 2009). The slower and more protracted aminoacidemia accompanying the ingestion of casein protein (Pennings et al., 2011), shown in pre-sleep protein ingestion studies (Res et al., 2012; Snijders et al., 2015), may be more effective at sustaining MPS and possibly at attenuating negative net protein balance (although all data to date on this mechanism are at the whole-body level) over longer periods of time. We propose the differences between protein sources in their ability to stimulate MPS are a combination of both the delivery (digestion) and AA composition of the protein, in particular leucine content. The AA composition in whey is superior to that of soy likely due to an increased leucine content (Tang et al., 2009). Lastly, there appears to be a leucine “threshold” for stimulation of MPS that is around ~3 g of leucine per meal (Churchward-Venne et al., 2014), which may be determining the per meal protein recommendation of ~0.4 g protein/kg.

## Protein and carbohydrate co-ingestion

The purpose of carbohydrate (CHO) co-ingestion with protein is to stimulate insulin release beyond that seen with AA ingestion alone with the idea that insulin improves net protein balance. Indeed, local insulin infusion at rest increases MPS (Biolo et al., 1995a, 1999; Hillier et al., 1998) and blood flow (Biolo et al., 1999). When insulin is infused along with AAs there is an increase in MPS (Bennet et al., 1990; Hillier et al., 1998) and slight attenuation of MPB (Bennet et al., 1990) beyond that of just AA ingestion (Bennet et al., 1990) or insulin infusion (Hillier et al., 1998). However, following RE, insulin infusion has no effect on blood flow or MPS, though the slight suppression of MPB remains (Biolo et al., 1999). Coinciding with the previous finding (Biolo et al., 1999), in response to a single bout of RE, the ingestion of CHO alone has no effect on MPS, but attenuates MPB (Roy et al., 1997; Børsheim et al., 2004). However, co-ingesting CHO with AA/protein following RE has no further stimulatory effects on MPS and does not suppress MPB so long as protein is adequate (~25 g) (Koopman et al., 2007; Glynn et al., 2010; Staples et al., 2011). These results indicate that when performing RE and providing adequate protein there is no benefit of co-ingesting CHO on stimulating MPS. This is most likely because the level of insulin required for optimal stimulation of MPS is remarkably low (Greenhaff et al., 2008; Trommelen et al., 2015) (i.e., 10–15 IU/ml), only 2–3 times basal resting levels for most healthy persons, which is easily reached with even a small dose of protein. With lower doses of protein (i.e., < 0.25 g protein/kg), however, CHO ingestion may impact net protein balance via the ability to increase systemic insulin and suppress MPB and/or enhance AA delivery to the muscle, but we need to experimentally test this thesis. We conclude that while ingestion of CHO post-exercise would be necessary to replenish depleted glycogen stores we do not see a strong need to recommend CHO on top of protein to be consumed post-exercise. It appears that even in a glycogen-depleted state protein is still effective at stimulating MPS following resistance exercise (Camera et al., 2012) and that only a minimal level of insulin is required to achieve optimal rates of MPS (Greenhaff et al., 2008).

## Training status

Training “age” may be an important variable impacting the quantity and duration of the anabolic response following RE. Compared to untrained participants, trained individuals have attenuated post-RE MPS and MPB resulting in less total muscle protein turnover (Phillips et al., 1999). A study by Tang et al. (2008) had participants train one leg for 8 week while the other served as the control. After the 8 week intervention, an acute bout of exercise stimulated a longer MPS response in the untrained or control leg relative to the trained leg suggesting an attenuation of the duration (but not magnitude) of MPS with training (Tang et al., 2008). Following a similar study design, after 8 weeks Kim et al. (2005) found an attenuation in mixed MPS in the trained leg, though myofibrillar protein synthesis remained the same. This finding is similar to that of Wilkinson et al. (2008) indicating a training-induced refinement, and perhaps efficiency, of post-exercise MPS. For a comprehensive review on the topic of training status and how it affects the MPS response and time course see Damas et al. (2015). The general conclusion from this review is that RE training reduces not the amplitude but the duration of the MPS response (Damas et al., 2015). This may in fact highlight that maximizing hypertrophic potential in the trained state may require greater focus on the post-exercise period for protein provision.

Despite the wealth of studies relating to the role of protein in augmenting the adaptive response to resistance exercise, relatively little has been conducted to identify whether resistance-trained individuals require greater relative post-exercise or daily protein consumption compared to those who are untrained. Data exist to suggest that athletes performing intensive periods of training may benefit from increased protein intake from the perspective of supporting immune function (Witard et al., 2014b). Moreover, those who engage in weight-categorized competition or sport may benefit from increased dietary protein intake (Mettler et al., 2010; Areta et al., 2014; Phillips, 2014b). However, as mentioned above, the post-RE MPS response reaches a maximum at 20 g or ~0.25 g/kg in both untrained (Moore et al., 2009) and trained (Witard et al., 2014a) young men. Whether or not these results hold true when performing whole-body RE has yet to be determined. We direct the interested reader to the following papers for more discussion on the topic: (Phillips and van Loon, 2011; Phillips, 2012, 2014b). The opinions of these reviews suggest that resistance-training athletes may benefit from larger protein intakes higher than the recommended dietary allowance in the range of 1.3–1.8 g/kg/day (Phillips and van Loon, 2011; Phillips, 2012, 2014b). Nonetheless, the training regimens of the modern athlete are often interdisciplinary in nature and it is therefore critical to appreciate the context of the research, athlete, and training paradigm before making recommendations regarding “optimal” protein intake. Regardless, consideration for the “3R” approach should be common practice.

## Resistance exercise program variables and training

Different skeletal muscle adaptations are induced by RE training than endurance training (Egan and Zierath, 2013). In this regard, we have shown that after 10 week of RE training, performing a single bout of RE increases myofibrillar, but not mitochondrial, protein synthesis whereas synthesis in both protein pools were acutely stimulated by RE in the pre-trained state (Wilkinson et al., 2008). Furthermore, with resistance training mixed MPS may decrease but fraction-specific adaptations (in this case myofibrillar MPS) may actually be enhanced (Kim et al., 2005). Indeed, it appears that the remodeling process following exercise is specific to the type of exercise performed (Wilkinson et al., 2008) and is tailored with training (Kim et al., 2005).

Manipulating different RE variables impacts both the acute and chronic anabolic response. For example, when young, resistance-trained (recreationally weight-training ≥2 times per week for ≥2 years) men received 20 g of whey protein after exercise, those who lifted with increased time under tensions (12 s per repetition) had elevated MPS compared to a repetition-matched control (2 s per repetition) (Burd et al., 2012). Specifically, Burd et al. (2012) found that sarcoplasmic MPS between 0 and 6 h, mitochondrial protein synthesis between 0–6 and 24–30 h, and myofibrillar protein synthesis between 24 and 30 h were all elevated with a longer time under tension beyond that of the repetition-matched group. It is worth noting that the repetition-matched group performing less time under tension per repetition lifted the same relative load. Indeed, the electromyography of the vastus lateralis indicated that the group exercising with a longer time under tension had increased muscle activity, and presumably muscle fatigue, toward the end of set completion (Burd et al., 2012). We speculate that the elevated MPS response to the longer time under tension is a result of increased motor unit recruitment which may be linked to muscle damage/remodeling (Proske and Morgan, 2001); however, we acknowledge we do not have experimental support for our proposed mechanisms. Interestingly, we have reported that when recreationally-active participants performed leg extensions at either 30 or 90% of their one-repetition max (1RM) to contractile failure there was an equal increase in mixed MPS (Burd et al., 2010). Additionally, 24 h after the RE bouts there was elevated myofibrillar MPS in only the 30% group (Burd et al., 2010). Not surprisingly, the 30% group had to perform more repetitions to achieve contractile failure and thus accumulated significantly more time under tension. Another study from our laboratory investigated this same principle over a 10 week period of training (Mitchell et al., 2012) in healthy but untrained young men and showed that the acute changes in MPS (Burd et al., 2010) mirrored those seen with training (i.e., equivalent hypertrophy). Though time under tension was not measured, it was concluded that regardless of the load lifted, performing RE to volitional failure results in hypertrophy (Mitchell et al., 2012). It appears that reaching contractile failure is required for optimal skeletal muscle growth. This can be achieved regardless of the repetition load. Manipulating variables such as time under tension or repetition-load may accelerate the time it takes to reach contractile failure by increasing muscle fatigue and enhancing the rate of motor unit recruitment, but they do not likely individually enhance MPS.

In contrast to current recommendations (American College of Sports Medicine, 2009), we propose that an important variable to consider in regards to the optimization of MPS and the subsequent hypertrophic response is to ensure, regardless of the load lifted, that loads are lifted to the point of contractile failure. Contractile failure, particularly when lifting lighter loads, often occurs when there is significant muscle fatigue and motor unit activation. Motor unit activation refers to the size and quantity of motor units recruited. The term “muscle fatigue” is frequently misinterpreted. Fatigue is the inability to produce maximal force; thus, muscle fatigue is the inability of recruited motor units to generate their maximal force output (Stephens and Taylor, 1972; Dorfman et al., 1990). Significant muscle fatigue is reached by activating and exhausting a full cadre of motor units (and thus fiber types) and, regardless of any RE variable, requires a high degree of effort. From a broad prescriptive standpoint, we have emphasized the need for a high degree of effort in performing RE (Phillips and Winett, 2010). We propose that the manipulation of a multitude of RE variables may mean much less in terms of stimulating hypertrophy than simply exerting a high degree of effort to achieve contractile failure.

**Figure 1:**
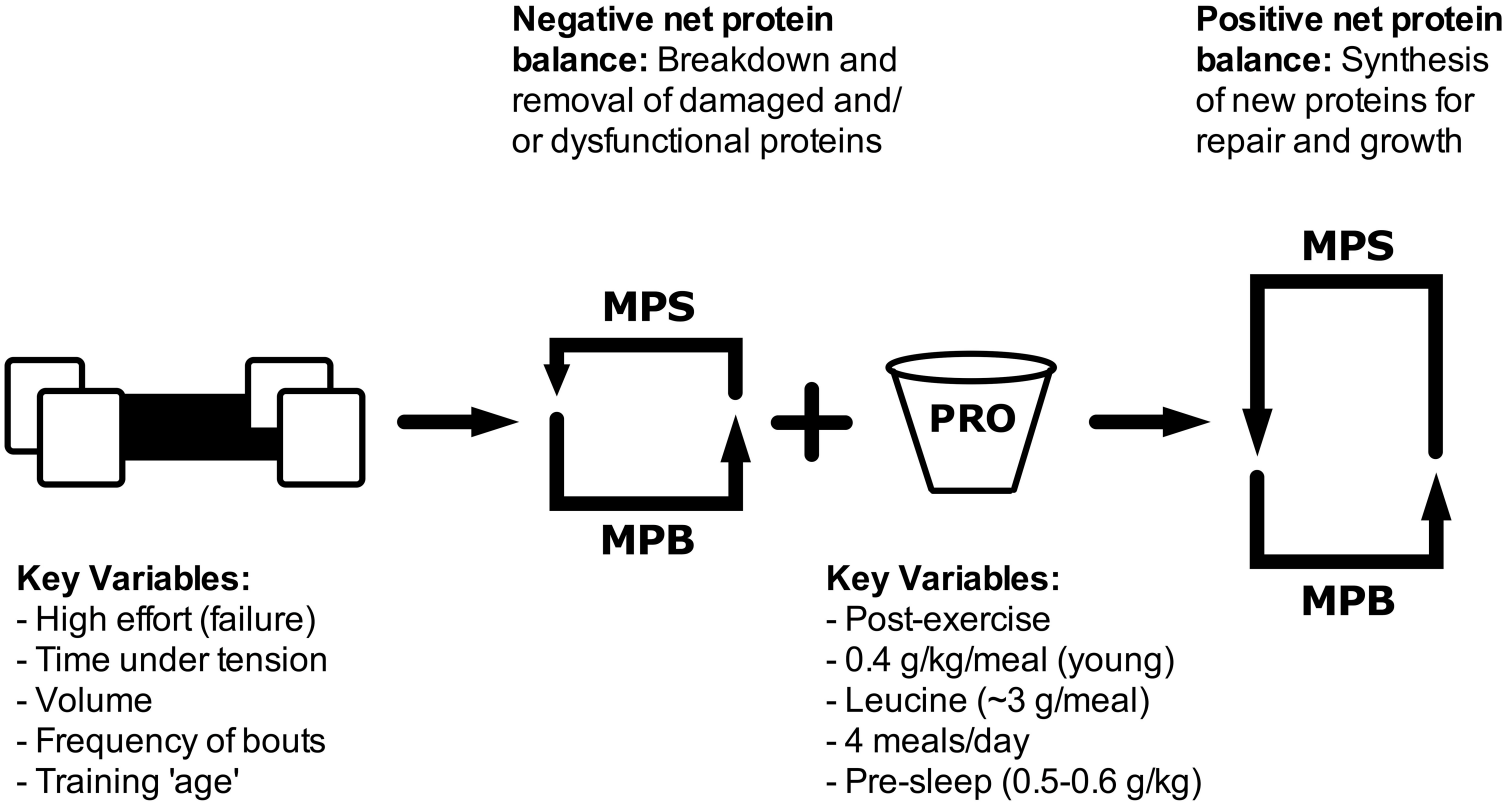
**Schematic showing how resistance exercise variables and protein ingestion can impact muscle protein turnover**. MPS, muscle protein synthesis; MPB, muscle protein breakdown; PRO, protein.

Relatively high (70–100% 1RM) training loads have been proposed to induce greater muscle hypertrophy (Campos et al., 2002; American College of Sports Medicine, 2009) than lower loads due to the increased mechanical loading and demand for fiber recruitment. However, as muscle fibers fatigue their motor units drop out and cease firing; a process that necessitates different motor units to be recruited to preserve the required force (Dorfman et al., 1990; Moritani et al., 1992). This is, at least partially, why surface electromyography and motor unit activation increase with muscular fatigue (Dorfman et al., 1990) and why similar hypertrophic adaptations are seen with varying external loads (Schoenfeld et al., 2014). Though lower loads may not initially need to recruit the larger motor units (innervating fast-twitch fibers) like higher loads may, with significant muscle fatigue there is an accompanied “dropout” of the smaller motor units (innervating slow-twitch fibers) such that subsequent contractions will be obliged to recruit additional (larger) motor units. If comparable motor units are activated and both groups are exercising until contractile failure it seems reasonable that similar adaptations are seen between low- and high-load RE training (Schoenfeld et al., 2014). However, we hypothesize that muscle fatigue (inability to generate maximal force) is not as important as motor unit activation in inducing muscle hypertrophy. For example, to reach contractile failure exercising at ~30% 1RM one would have to achieve ~70% muscle fatigue. In contrast, to reach contractile failure at ~70% 1RM, an individual would only achieve ~30% muscle fatigue. Thus muscle fatigue, albeit rendering an increase in motor unit activation, cannot be the most important determinant of the skeletal muscle response to RE if low- and high-load RE are inducing similar MPS (Burd et al., 2010) and hypertrophy (Mitchell et al., 2012). Instead, we hold on to the hypothesis that reaching contractile failure is what drives skeletal muscle adaptation (see Figure 1). We emphasize that it is naïve to prescribe moderate-heavy loads as the only way to induce muscle hypertrophy (American College of Sports Medicine, 2009). We also acknowledge that, as Mitchell et al. (2012) has shown, there may be a neuromuscular effect where the practice of lifting heavier loads over longer durations stimulates greater improvements of muscular strength. This is potentially due to a lack of inhibition on afferent feedback (Amann et al., 2009), but future research is required to be certain.

A number of meta-analyses on the impact of different RE program variables on muscle strength and hypertrophy are available (Peterson et al., 2005; Krieger, 2010; Schoenfeld et al., 2014, 2015). The conclusion, on examination of these analyses (Peterson et al., 2005; Krieger, 2010; Schoenfeld et al., 2014, 2015), would be that exercise volume (load × sets × reps) and training frequency (sessions per week) are important variables that affect the hypertrophic response and to this list we would propose the addition of effort. Contrary to popular belief, muscle hypertrophy may not be significantly influenced by resistance exercise load (Schoenfeld et al., 2014). This is despite 7 out of the 11 studies being volume equated, essentially suggesting the participants in the low-load groups did not train until contractile failure (Schoenfeld et al., 2014). We recognize there are many other variables that are manipulated to maximize changes in muscle mass, however, we hypothesize that these are largely moot when contractile failure is reached. Instead of any particular medley of RE variables, we propose that muscular hypertrophy is fundamentally driven by maximal motor unit recruitment and exercising until contractile failure.

## Funding

SMP gratefully acknowledges funding from the National Science and Engineering Council of Canada (RGPIN-2015-04613) and the Canadian Institutes for Health Research (MOP-123296) as well as the Canadian Diabetes Association (OG-3-14-4489).

### Conflict of interest statement

Stuart M. Phillips declares that he has received honoraria and travel expenses from the US National Dairy Council, Dairy Farmers of Canada, and the US National Beef Cattlemen's Association. Stuart M. Phillips, Robert W. Morton, and Chris McGlory declare no conflicts of interest financial or otherwise in conjunction with the writing of this paper.
